# Chikungunya Disease: Infection-Associated Markers from the Acute to the Chronic Phase of Arbovirus-Induced Arthralgia

**DOI:** 10.1371/journal.pntd.0001446

**Published:** 2012-03-27

**Authors:** Laurence Dupuis-Maguiraga, Marion Noret, Sonia Brun, Roger Le Grand, Gabriel Gras, Pierre Roques

**Affiliations:** 1 CEA, Division of Immuno-Virologie, Institute of Emerging Diseases and Innovative Therapies, Fontenay-aux-Roses, France; 2 UMR E1, University Paris Sud 11, Orsay, France; Centre for Cellular and Molecular Biology (CCMB), India

## Abstract

At the end of 2005, an outbreak of fever associated with joint pain occurred in La Réunion. The causal agent, chikungunya virus (CHIKV), has been known for 50 years and could thus be readily identified. This arbovirus is present worldwide, particularly in India, but also in Europe, with new variants returning to Africa. In humans, it causes a disease characterized by a typical acute infection, sometimes followed by persistent arthralgia and myalgia lasting months or years. Investigations in the La Réunion cohort and studies in a macaque model of chikungunya implicated monocytes-macrophages in viral persistence. In this Review, we consider the relationship between CHIKV and the immune response and discuss predictive factors for chronic arthralgia and myalgia by providing an overview of current knowledge on chikungunya pathogenesis. Comparisons of data from animal models of the acute and chronic phases of infection, and data from clinical series, provide information about the mechanisms of CHIKV infection–associated inflammation, viral persistence in monocytes-macrophages, and their link to chronic signs.

## Introduction

The major epidemic of chikungunya virus fever (chikungunya) that affected 266,000 people in La Réunion in 2006 highlighted the vulnerability of immunologically naive populations and raised interest in this disease in the media, governments, and the scientific community [1]. Over and above the number of cases, with one-third of the population affected, this epidemic revealed new features of chikungunya pathogenesis.

An adaptive mutation in the virus led to *Aedes albopictus* becoming a major vector for chikungunya virus (CHIKV) [2], resulting in a risk of disease in previously unaffected areas. In addition, other new or previously underestimated features of the virus have also come to light in recent chikungunya epidemics. Atypical symptoms were observed in the La Réunion epidemic (Table 1), albeit in a small percentage of patients (<0.3% [3], [4]). However, more importantly, this epidemic highlighted the persistence of chronic features in a significant proportion of patients, as first reported in the early 1980s [5]–[9] but not investigated further. These chronic signs of chikungunya are disabling and merit further attention, because there is currently no recommended treatment for chikungunya based on clinical trials [10]–[13]. Treatment instead depends on the response of the patient, ranging from non-steroidal anti-inflammatory drugs, to re-education, to simple rest [14], [15].

**Table 1:** Acute chikungunya symptoms in typical and atypical forms in adults, children, and newborns.

Setting	La Réunion and Indian Ocean Islands [3], [4], [14], [21], [22], [67]–[69]	India [16], [19], [27], [70]–[76]	Singapore [77]	Malaysia [78], [79]
***Mean age (year)***	34±20	35 (median)	37(median)	35.9±18
***Sex ratio (male/female)***	0.84	0.6 – 0.9	3.4	0.82 – 1.03
***Typical form symptoms % (common before 2006)***				
Fever	100	81 – 100	90	86 – 100
Arthralgia *joint pain*/*swelling*	100	86 – 80	88	82 – 100
Cephalalgia *headache*	70	93	43	30 – 50
Myalgia *muscle pain*	64	48 – 84	61	10 – 48
Rash	39	36 – 50	36	64 – 17 – 50
Erythema	33	—		
Asthenia *fatigue*	67	43		
***Atypical Symptoms % (as defined in La Réunion; *** [***3]***, [4** ****] ***)***	*n* = 876; atypical form is 36% of severe acute chikungunya but less than 0.3% total cases in La Réunion			
Maculopapular eruption	33	17		
Meningo-encephalitis	16	25		
Diarrhea / vomiting	18	12/4	3/8	
Renal failure	20	—		
Respiratory failure	16	—		
Myocarditis / pericarditis	6	11		
Hepatitis	6	—		Yes^a^ [80]
Sensory changes (muffling of noise, sensitivity to light)	9	33		
Aphtous-like ulcer	—	21		
Hyperpigmentation	15	20		
Genital ulcers	—	1		
Optic neuritis	<1	<1	11^b^	
Facial paralysis	—	2		
Guillain-Barre syndrome	1	Yes^a^ [81]		
***Atypical symptoms in young children %***	[3], [23], [82]	[72], [75]		
Hyperalgia *high grade pain*	27	Yes^a^		
Diarrhea/vomiting	27	Yes^a^		
Convulsions	22	Yes^a^		
Bullous dermatosis	17	Yes^a^		
Purpura	8	Yes^a^		
Encephalitis	6	Yes^a^		

a Atypical forms were noticed but their prevalence was not evaluated.

b Retro-orbital pain.

We aim here to present current knowledge on CHIKV infection and related aspects, such as the inflammatory response which potentially leads to the development of chronic syndromes.

## Clinical Symptoms, from Acute to Chronic Chikungunya

**Figure 1:**
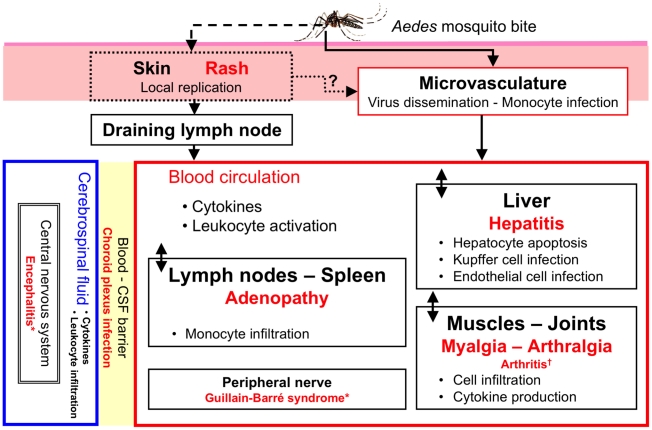
Virus dissemination and target organs. Following inoculation with CHIKV through a mosquito bite, the virus directly enters the subcutaneous capillaries, with some viruses infecting susceptible cells in the skin, such as macrophages or fibroblasts and endothelial cells. Local viral replication seems to be minor and limited in time, with the locally produced virus probably being transported to secondary lymphoid organs close to the site of inoculation. The blood carries most viruses, as free virions or in the form of infected monocytes, to the target organs, the liver, muscle, joints, and remote lymphoid organs. In these tissues, infection is associated with a marked infiltration of mononuclear cells, including macrophages, particularly when viral replication occurs. The pathological events associated with tissue infection are mostly subclinical in the liver (hepatocyte apoptosis) and lymphoid organs (adenopathy), whereas mononuclear cell infiltration and viral replication in the muscles and joints are associated with very strong pain, with some of the patients presenting arthritis. * Guillain-Barré syndrome and encephalitis are very rare events. † True arthritis remains a rare event (from 2% to 10%); see Table 2.

CHIKV spreads rapidly in the body after initial infection (Figure 1). The clinical signs of acute infection are not entirely specific and may vary between cohorts (Table 1). A small but significant number of infected people present asymptomatic infection (5% to 18% [16]–[18]), significantly more often below 25 years [19], [20]. However, concomitant fever and arthralgia is a common specific sign [17], [20]–[22]. The La Réunion epidemic highlighted various “new” symptoms considered atypical and frequently associated with severe forms, other than in very rare cases of vertical transmission (Table 1) [23]. Most of these symptoms were also observed in India and in travelers returning from areas of endemic disease [7], [24]–[26].

Acute-phase symptoms usually disappear after 2 weeks. However, arthralgia and/or myalgia may persist for weeks, months, or even years. Some patients go on to develop a genuine, chronic arthritic syndrome. Typically, joint damage fluctuates over time, but always affects the same parts of the body, mostly the extremities (hands, ankles, knuckles; Table 2) [5], [7], [8], [26], [27]. Chronic chikungunya has been described before, but the La Réunion epidemic brought it to public attention. In 1979, Fourie and Morrison described a rheumatoid arthritic syndrome affecting 18% of patients in South Africa [9]. Their observations were confirmed in 1983 by Brighton et al., who reported that 12% of patients with CHIKV in South Africa continued to display rheumatic manifestations years after the acute phase [6]. Being over the age of 40 years was identified as a risk factor for chronicity. This rheumatic syndrome may reflect the ability of CHIKV to persist in some compartments by mechanisms that remain poorly understood.

**Table 2:** Chronic chikungunya symptoms.

Setting (Date Referred)	La Réunion (2005 to 04/2006) [5]	La Réunion (04/2006–2007) [41], [83]	India Karmataka (2008) [27]	India Maharashtra (2006) [19]	Singapore (2008) [44], [77]
**Number of chronic cases / number of patients in the cohort ** ***(month after acute phase)***	56^a^/88 *(>18)*	116/133 *(12)*	94/203 *(>9)*	59/315 *(12)*	14^b^/97 *(3)*
**Mean age of chronic cases**	60	58 (median)	41	48	38±6
**Sex ratio (M/F in chronic)**	0.93^c^	0.33	0.81	0.33*	1.8
**Hospitalized (%)**	65.9	85	0	0	100
**Joint pain (%)**	100	100	94	100	100
**Fatigue (%)**	NR	NR	27	NR	NR
**Joint swelling/tenosynovitis (%)**	16	NR	58.5	NR	NR
**Localization**					NR
**Symmetrical (%)**	64	More often	83	NR	
**Knee (self/exam) (%)**	21 (57)	20	59.5	57	
**Ankles/wrist (%)**	29/16	31/21	54/31	15/59	
**Shoulder (%)**	30	NR	15	52.5	
**Hips (%)**	5	NR	11	7	
**Continuous pain (%)**	55	68		NR	100
**Rheumatoid like polyarthritis (%)**	ND	10	36^d^	100 NSA	0
**Spondylo-arthropathy (%)**	ND	22	IE	7 IA	0
**Underlying disease (%)**	71	Not detailed	10/20 tested by MRI	NR	0
**Previous arthritis/arthralgia (%)**	51	7	7	5	0

*: *p*<0.01 Khi-2 between infected time 0 and chronic at 12 months (calculated by us).

a Low ALT/ASAT level related to persistence (*p*<0.01) like platelet count.

b Lower level of creatinine *p* = 0.036.

c No difference chronic versus recovered.

d American College of Rheumatology criteria for rheumatoid arthritis in [28]; note that tenosynovitis is prominent (as seen by Hoarau et al. [41]).

IA, inflammatory arthritis; IE, inflammatory, erosive; MRI, magnetic resonance imaging; ND, not detected or prevalence not evaluated; NR, not reported; NSA, non-specific arthritis.

Following the 2005–2006 epidemic in La Réunion, 36% of patients reported the persistence of symptoms 15 months after disease onset, and 21% reported at least one recurrence [8]. In this group, age over 45 years, the pain intensity score (≥7 on a 0–10 scale) during acute disease, and pre-existing osteoarthritis conditions were associated with persistence. However, classification was based on self-perceived recovery from rheumatic manifestations of chikungunya, and it is therefore very difficult to differentiate between osteoarthritis and chikungunya in these patients (Table 2). Nevertheless, age and pain intensity score remain robust predicting factors. Similar chronic forms of chikungunya had previously been reported in other patient series in La Réunion by Borgherini et al. [5] and in travelers returning from Indian Ocean islands by Simon et al. [7]. These late signs may not be specific to the CHIKV strain of the 2005–2006 outbreak, as they were also reported for a closely related viral strain in India, with various frequencies in the 10 months following the acute phase: 16% in Maharashtara and 49% in Karnataka [19], [27] (Table 2). In Karnataka, Manimunda et al. examined 20 of 94 patients with persistent joint pain by X-ray and MRI: 15% of patients presented chronic inflammatory erosive arthritis after CHIKV infection [27]. By contrast, Chopra et al. mostly reported chronic pain, with rare cases of inflammatory arthritis, in a population cohort study; these findings conflict with their previous report on patients hospitalized at a rheumatology referral center [19], [28].

Persistent symptoms, including arthralgia, myalgia, and arthritis (consistently found on radiological/MRI examination in various sets of patients, see Table 2 and [7], [26]–[29]) suggest the persistence of virus in target organs or the establishment of a self-sustained, deleterious mechanism leading to tissue damage, probably inflammatory in nature. Very little is currently known about the mechanism underlying chikungunya persistence. Studies in animal models and clinical investigations are needed to address this point.

## Animal Models

The mouse was the first organism used for *in vivo* studies of CHIKV infection [30], but muscle and joint disease was achieved only recently in this species [31]–[33]. Subcutaneous CHIKV inoculation close to distal leg joints in adult C57Bl6 mice leads to a muscle and joint disease, including loss of balance, hind limb dragging, and skin lesions [31], [32], [34]. This strongly suggests that the initial dissemination of CHIKV (Figure 1) is critical to the setting of chikungunya. In this model, and using both Asian and La Réunion strains of CHIKV, Gardner and Morrison demonstrated arthritis, tenosynovitis, and myositis, together with local virus persistence in tissues after the phase of active replication in the blood. Infiltration with monocytes and macrophages is also observed in connective tissues, and subcutaneous peritendinous tissues and muscle, on histological analysis and immunohistochemistry with the F4/80 monoclonal antibody [31], [33], [34]. In 2006, we developed a model of macaque infection and virus spread (Figure 1). This model was required for the rapid implementation of preclinical trials following the La Réunion epidemic and the risk of extension to the Indian Ocean zone. We infected cynomolgus macaques (*Macaca fascicularis*) with CHIKV strain LR-2006-OPY1 from La Réunion [29]. RT-PCR on plasma revealed viral replication kinetics similar to those in humans, together with profound leukopenia consistent with lymphocyte and monocyte recruitment to tissues. Several soluble factors, such as IFNα, IL-6, MCP1/CCL-2, and TNFα, were found to be induced, to various levels, in a multiplex immunoassay [35]. In this model, acute infection is controlled by the immune response, which renders viral replication undetectable, from 10 days after infection [35]. However, CHIKV persists in target tissues after its clearance from the blood, as demonstrated by viral RNA detection in an *in situ* hybridization assay using a CHIKV subgenomic mRNA specific probe. Joints, secondary lymphoid organs and, to a lesser extent, muscles, are affected. CHIKV replicates in several cell types during the acute phase [35], but is thereafter detectable only in macrophages, by immunohistochemical analysis with a monoclonal antibody against the viral E2 glycoprotein. We detected infected monocytes-macrophages in the blood 6 hours after infection (flow cytometry) [36] and in most tissues on the following day (*in situ* hybridization, immunohistochemistry, RT-PCR, and virus isolation). Significant macrophage infiltration was also detected by histological analysis [35], throughout the study and long after viral clearance from blood. This monocyte-macrophage tropism is consistent with recent findings from Lisa Ng's team that human monocytes are susceptible to infection *in vitro*, as shown by flow cytometry with a monoclonal antibody against the E1 glycoprotein of CHIKV. Infected monocytes generate new viruses, which can be detected with HEK293 cells and titration assays, albeit at low levels [37]. Similarly, CHIKV can infect primary macrophages *in vitro* [38], [39], resulting in the production of highly variable amounts of virus, from 10^3^ to 10^6^ pfu per ml [31], [35], [39], [40]. These results are consistent with human studies reporting that macrophages are susceptible to CHIKV infection both *in vivo* and *in vitro* [41]–[43]. The alleviation of chikungunya-associated arthritis and myositis by treatment with the MCP-1/CCL-2 inhibitor Bindarit in mice [33] also strongly suggests that monocytes-macrophages, the main targets in MCP-1/CCL-2 tissue tropism, are central to muscle and joint disease.

There is currently no animal model reproducing the chronic rheumatoid syndrome of chikungunya. Indeed, the disease has too short a course in mice [31], [32], and no joint damage was evident in macaques [35], in which only virus persistence in joints, long after the acute phase, could be demonstrated. Nevertheless, both models suggest that inflammation, macrophage tissue tropism, and local viral persistence are involved in the establishment of chronic disease.

## Viral Persistence and Clinical Expression

**Figure 2:**
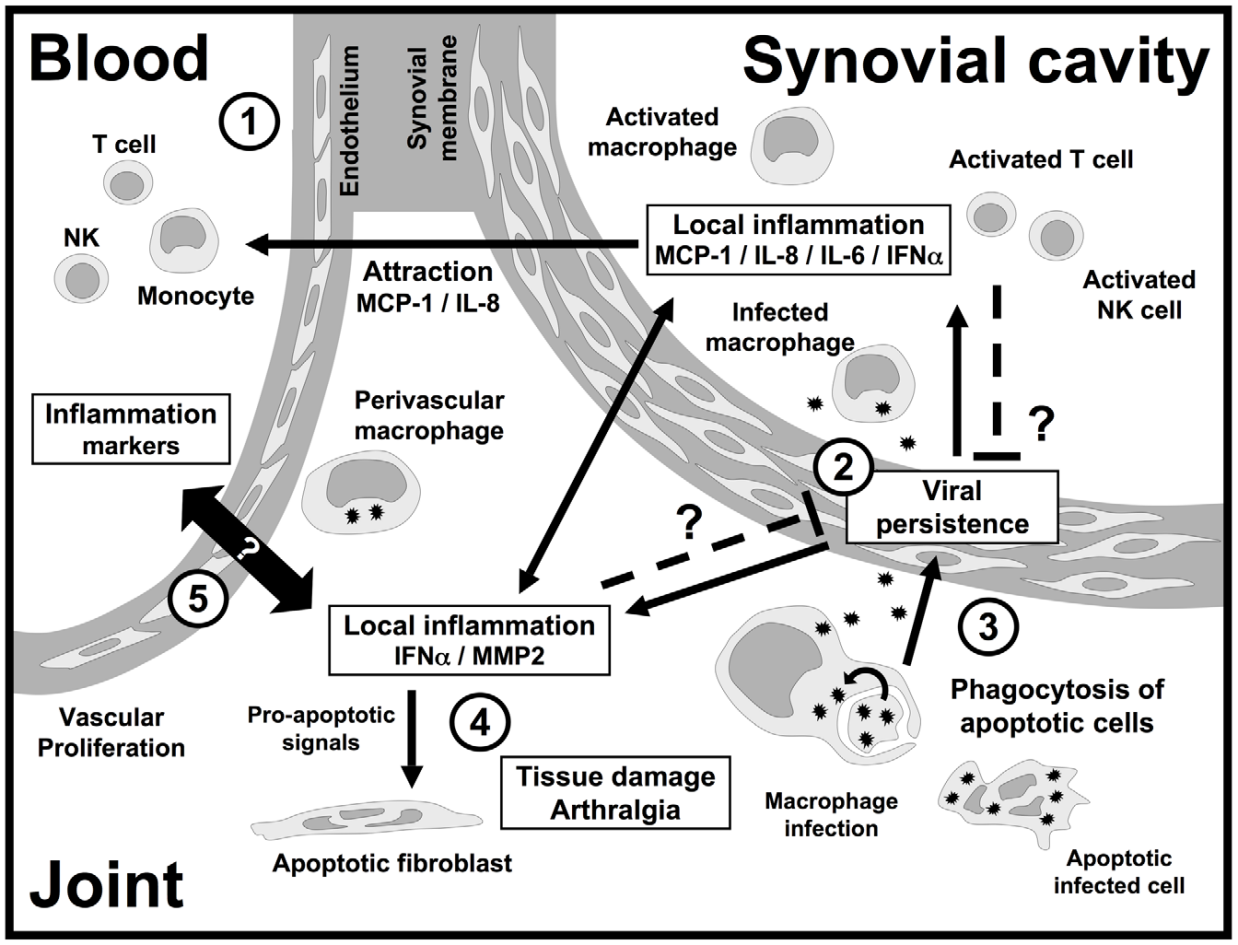
Mechanisms of CHIKV persistence and tissue inflammation in patients with chronic disease. (1) Months after the acute infection, monocytes, T cells, and natural killer (NK) cells are still attracted to the inflamed joint, where they become activated. (2) The infection of macrophages in joints is associated with local inflammation and the production of cytokines, chemokines, and pro-inflammatory effectors, such as MCP-1/CCL-2, IL-8, IL-6, IFN-α, and MMP2. (3) The phagocytosis of apoptotic bodies from infected cells probably contributes to viral persistence. Nevertheless, the beneficial or deleterious effect of local inflammation on viral persistence remains unclear. (4) When it occurs, arthritis is accompanied by high rates of fibroblast apoptosis and cartilage destruction. Chronic inflammation probably plays a major role in this damage and associated pain. (5) The potential relationship between local inflammation of the joint and a state of systemic activation, as demonstrated by the presence of inflammation markers in plasma and blood cells, remains unclear.

Recent data have clearly implicated inflammatory mediators not only in the acute and resolution phases of chikungunya, but also in the establishment of chronic disease. Figure 2 shows how CHIKV infection may lead to chronic joint damage.

### The Acute Phase

The first phase of CHIKV infection is typical of acute viral infection, with a very early type 1 interferon (IFN) response [41], [44]–[46]. IFN-α is detected on the first day of infection and its concentration is correlated with plasma viral load, which is significantly higher in elderly patients [47]. However, plasma concentrations of Th1 and Th2 cytokines remain low [41], [44]–[46], [48], despite the observation by Hoarau et al. that IFN-γ and IL-12 levels were high in patients from La Réunion [41]. Wauquier et al. also reported IFN-γ overproduction, together with IFN-α2, during the acute phase [45], in Gabon. Chow et al. showed, in Singapore, that IFN-α was produced for 10 days before viral clearance from plasma, but they detected no IFN-γ in serum [44]. The acute IFN response may be short-lived, as it has not been detected directly in studies otherwise reporting an early increase in levels of the IFN-inducible chemokines MIG/CXCL-9 and IP-10/CXCL-10) [46], [48].

Viral load is related to the concentrations of IFN-α, IL-1-RA, IL-6, MCP-1/CCL-2, IL-12 and IP-10/CXCL-10 [44], IL-18, and IL-18BP [49]. The inflammatory response to CHIKV infection therefore clearly contributes to virus elimination. Unfortunately, none of the population studies [19], [27], [41], [44]–[46], [48] carried out assessed joint or muscle pain, so it is not currently possible to determine the relationship between viral load or inflammatory mediators and symptoms. Wauquier et al. reported markedly higher levels of proinflammatory mediators than other teams [41], [44]–[46], [48]. This difference may reflect regional characteristics of the cohorts studied (Gabon versus Italy, La Réunion, India, and Singapore), with different genetic backgrounds and heterogeneous sanitary conditions, but it may also reflect sampling schedule. For example, IL-6 concentration depends largely on sampling time, as observed in our macaque model [35]. Similarly, IL-12 concentration was high in the Gabon, La Réunion, and Singapore series [41], [44], [45], but was lower than that in uninfected controls in Italy, although these results may not be directly comparable due to the use of different technologies [46]. During the experimental infection of macaques with CHIKV, we observed an early induction of IFN-α, MCP-1/CCL-2, and IL-6, followed by the detection of MIP-1α/CCL-3 and MIP-1β/CCL-4, IFN-γ, and TNF-α. These last two effectors remain at higher concentrations than in controls until the end of the recovery phase (15–20 days post infection) [35]. In the mouse model developed by Surhbier et al., events follow a similar chronology, but with differences in plasma cytokine concentrations, including higher levels of TNF-α [31].

### The Chronic Phase and Its Predictive Factors

The inflammatory response to CHIKV infection leads to viral elimination from the blood and clinical recovery. However, it is now clear that disease may persist in a subgroup of patients presenting variable levels of myalgia and arthralgia, culminating in some cases in a debilitating arthritic syndrome. Five studies have tried to identify the factors associated with chronic chikungunya disease in groups of patients in Singapore [44], La Réunion [41], Dakshina Kannada (India) [27], [48], and Emilia Romagna (Italy) [46] (Table 3), with times measured from inclusion (i.e., first consultation).

**Table 3:** Biological parameters associated with disease chronicity in chikungunya patients.

Parameter	Recovered Patients	Chronic Patients	α Risk	Series
**Age**	50.3±13.7	70.7±15.5	2%	La Réunion [41]
	40.2±12.3	31.5±5.2	NS	Singapore [44]
	34.5	41.5	NS	Dakshina Kannada [48]
	Cure rate 10 months after inclusion declining with age		<1‰	Dakshina Kannada [27]
**Acute phase**				
Viral load at inclusion	2.3×10^6^±3.7×10^6^ cp/ml	3.9×10^9^±6.9×10^9^ cp/ml	5‰	La Réunion [41]
	6.9×10^7^±1.5×10^8^ pfu/ml	1.12×10^4^±5.2×10^3^ pfu/ml	NS	Singapore [44]
Plasma CRP concentration at inclusion	11.33±10.05 mg/ml	60.2±59.7 mg/ml	7%	La Réunion [41]
Circulating cytokine concentrations at inclusion	Th1 and Th2, moderate	Trend toward a Th1 bias	NS	La Réunion [41]
**Recovery phase**				
Circulating GM-CSF, 2–3 months post inclusion	Normal	Higher than controls	<5%	Singapore [44]
Circulating eotaxin and HGF, 2–3 months post inclusion	Higher than controls	Normal	<5%	Singapore [44]
Circulating IL-6, 2–3 months post inclusion	Lower than controls	Normal	<5%	Singapore [44]
**Chronic phase**				
Circulating IL-12 p40 post acute phase	Not detectable	Persistent (±1 ng/ml)	0.5‰^a^	La Réunion [41]
Circulating MIG/CXCL-9 and IP-10/CXCL-10, 6 months post inclusion	Higher in patients with chronic disease than in fully recovered patients		<5%	Emilia Romagna [46]
CHIKV-specific IgG titer, 6 months post inclusion	Higher in patients with chronic disease than in fully recovered patients		<5%	Emilia Romagna [46]
Circulating cytokines, 10–12 months post inclusion	Th2 (increased IL-5)	Th1: IL-1β, IL-1RA, IL-6, MCP-1/CCL-2, MIP-1α/CCL-3, MIP-1β/CCL-4	<5%	Dakshina Kannada [48]
	No difference between patients with chronic disease and fully recovered patients		NS	Emilia Romagna [46]
IFN-α mRNA in PBMC, 12 months post inclusion	Not detectable	High levels	2%^a^	La Réunion [41]
**Joint tissue**				
CHIKV detection in the joint at late time points (>12 months)	No	Yes, in macrophages	NA	La Réunion [41]
Joint inflammation at late time points (>12 months)	No	MCP-1/CCL-2, IL-8, IL-6, MMP2, IFN-α	NA	La Réunion [41]

a Mann and Whitney test performed by us.

Inclusion: first presentation of the patient during acute disease.

Numbers (and sex ratios) of patients with chronic disease were 5 (4) in Singapore, 32 (0.15) in La Réunion, 94 (0.82) in Dakshina Kannada, and 35 (sex ratio not available) in Emilia Romagna.

cp, viral RNA copies; CRP, C reactive protein; NA, not applicable; NS, not significant; pfu, plaque-forming units.

The proportion of patients with chronic signs attributable to CHIKV differed between series. In Singapore, 13% of the infected patients still had chronic arthralgia 3 months after infection [44]. In Emilia Romagna,70% of patients had symptoms persisting at 6 months and 32% had symptoms persisting at 12 months after inclusion [46]. Similarly, 49% of patients in the 2008 outbreak in Dakshina Kannada still had symptoms attributable to chikungunya 10 months after inclusion [27], and almost half the studied patients from La Réunion had persistent symptoms, possibly including arthritis, 1 year after inclusion [41].

Patients with chronic symptoms differ considerably in acute infection characteristics. Hoarau et al. reported high baseline viral loads in patients with chronic disease (10 versus 2.1×10^9^ copies/ml, *p* = 0.005), whereas the opposite trend was reported in Singapore. Viral load was not considered in the Emilia Romagna and Dakshina Kannada series.

Patient age appeared to be crucial in the La Réunion and Dakshina Kannada groups, but not in Singapore (see Table 3). Patients with chronic chikungunya were older than those who recovered fully in La Réunion [41], whereas cure rate at 10 months decreased with age in Dakshina Kannada [27].

Hoarau et al. also suggested a link between chronic disease and a stronger inflammatory Th1 response to acute infection (Table 3). Patients with chronic disease displayed stronger systemic inflammation during the acute phase (higher CRP levels, *p* = 0.07), potentially associated with higher levels of TNF-α, IL-8, IL-6, and IL-12, although these differences were not significant [41]. Conversely, the Th2 cytokines IL-4 and IL-13 tended to be produced in smaller amounts during the acute phase in patients progressing to chronic disease.

**Figure 3:**
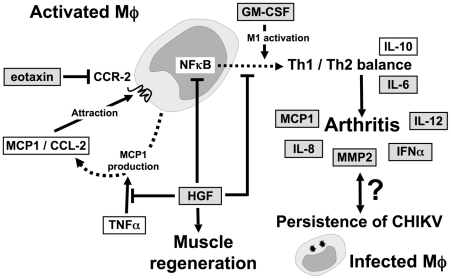
The macrophage is central to chronic signs of chikungunya disease. Macrophage infiltration, under the control of MCP-1/CCL-2, is a critical feature of damaged tissues. The inflammatory effectors IL-6, IL-8, MCP-1/CCL-2, MMP2, and INF-α are specifically expressed in the tissues of patients with chronic chikungunya, who have high IFN-α and IL-12 mRNA levels in their circulating leukocytes. This classical inflammatory process may be regulated by HGF and eotaxin, which have different expression profiles during the recovery phase in patients with chikungunya, depending on whether or not these patients go on to develop chronic disease. HGF also promotes muscle regeneration. Once they have infiltrated the joint or muscle, the macrophages are activated and regulate the local Th1/Th2 balance as a function of their own activation status (classical/M1 or alternative/M2). GM-CSF and HGF, which have M1 and M2 effector activities, respectively, may modulate this balance as they are differentially expressed in acute and chronic chikungunya. CHIKV persists in infected macrophages only in patients with a chronic rheumatic syndrome. The reciprocal influences connecting viral persistence and local inflammation are not known. Solid arrows: activation. Solid stopped lines: regulation. Dotted arrows: expression.

The recovery period lies between the acute and chronic phases. During this period, active regulatory mechanisms responsible for the resolution of inflammation take place. These mechanisms mostly involve macrophages and their unique ability to arouse and regulate inflammation (Figure 3). Chow et al. distinguished early (4 days after inclusion) and late (10 days after inclusion) convalescent phases, whereas Hoarau et al. measured cytokine levels 15 days after inclusion, corresponding roughly to the late convalescent phase. Chow et al. reported the upregulation of both pro-inflammatory and regulatory mediators (IL-2R, IL-4, MIG/CXCL-9, MIP-1α/CCL-3, hepatocyte growth factor [HGF], basic fibroblast growth factor [bFGF], granulocyte colony-stimulating factor [G-CSF], and eotaxin/CCL-11) during the early phase of convalescence. During the late convalescent phase, epidermal growth factor [EGF] and RANTES/CCL-5 concentrations peaked in patients from Singapore and IFN-γ and IL-12 were overproduced in patients from La Réunion.

The recovery phase appears to play a critical role in the establishment of chronic disease, at least in patients from Singapore. Chow et al. found higher GM-CSF (granulocyte-macrophage colony-stimulating factor) concentrations in patients subsequently displaying chronic symptoms than in those who fully recovered and the control group [44]. This is consistent with the role of GM-CSF as a proinflammatory mediator in rheumatoid arthritis [50], [51] and in the activation of monocytes-macrophages [52]. Conversely, during convalescence, cured patients continued to have significantly higher levels of HGF and eotaxin/CCL-11 than control subjects and patients with chronic pain syndrome. Eotaxin is a Th2 chemokine and natural antagonist of CCR-2, the receptor for MCP-1/CCL-2 [53], whereas HGF inhibits the production of MCP-1/CCL-2 in response to TNF-α *in vitro* [54]. The concomitant overproduction of eotaxin and HGH therefore probably reflects the inhibition of MCP-1/CCL-2 signaling via CCR-2. MCP-1/CCL-2 is a major chemoattractant for monocytes-macrophages. It is strongly expressed during acute infection in humans and in animal models [31], [35], [46], [48], [55], [56]. It is the target of the pharmacological inhibitor Bindarit, a molecule that alleviates chikungunya arthritis and myositis in the mouse model [33]. During recovery, plasma MCP-1/CCL-2 concentration decreases, remaining slightly (and non-significantly) above normal levels 2–3 months later. The high levels of eotaxin and HGF production in patients attaining full remission [44], and the beneficial effects of CCR-2 inhibition in the mouse model [33], strongly suggest that recovery requires the inhibition of signaling downstream from CCR-2, preventing monocyte-macrophage recruitment to tissues (Figures 2 and 3).

HGF also has beneficial effects on muscle regeneration [57]. It regulates the IL-6/IL-10 balance in favor of IL-10 in various models [54], [58], [59], probably by inhibiting NFκB [60]. HGF overproduction may therefore account for the low IL-6 concentrations in patients fully recovering in Singapore and the small number of severe cases in this group. A Th2-type response in patients that recovered (10 months after inclusion) was also described by Chaaithanya et al. [48]. Conversely, a deficit of HGF may account for the persistence of high IL-6 levels in the chronic phase, both systemically [19], [48] and locally [41]. IL-6 is involved in joint pain [61] and rheumatoid arthritis [62], and it increases the production of cartilage-destroying enzymes [63].

In addition to IL-6, chronic chikungunya (>6 months) is also associated with other systemic markers of inflammation, such as IFN-α [41], the IFN-inducible chemokines MIG/CXCL-9 and IP-10/CXCL-10 [46], and the cytokines IL-1β, IL-1RA, IL-6, CCL-2/MCP-1, CCL-3/MIP-1α, CCL-4/MIP-1β [48], IL-12 [41], and IL-17 [44]. Th1-type activation has also been observed in altered joint tissues, in which it is associated with viral persistence in macrophages [41] (Figure 2).

These data highlight the role of macrophages in chronic arthralgia and arthritis as a virus reservoir and as the main local cell type involved in regulating inflammation and Th1/Th2 balance. The central role of macrophages in chronic chikungunya disease and various pathogenic pathways involved are illustrated in Figure 3.

## Conclusion

Regulatory mechanisms seem to be required to prevent the establishment of chronic disease weeks or even months after viral clearance from the blood. The absence of such mechanisms leads to chronic arthralgia, or the arthritis observed in the group from La Réunion. Hoarau et al. detected CHIKV and various markers of inflammation (IFN-α, IL-6, MCP-1/CCL-2, IL-8, and MMP2) in the synovium of a patient suffering from chronic pain, but not in synovium of two patients who recovered fully [41]. The persistence of a local reservoir of CHIKV in joints may therefore be characteristic of chronic disease (see Figures 2 and 3). This persistence is consistent with findings in the macaque model, in which CHIKV is detected after up to 90 days in tissues, including joint tissues [35].

The persistence of CHIKV in joints may therefore lead to chronic local inflammation, causing pain. Local inflammation would in turn establish local conditions favoring CHIKV persistence. Indeed, we know from HIV/AIDS studies [64], [65] that inflammation contributes to the destruction and elimination of viruses, but may also maintain the levels of activation required for sustained replication and viral persistence in tissues, particularly in macrophages, a cell target common to HIV and CHIKV. Thus, CHIKV persistence may therefore also result from an imperfectly resolved inflammatory phase, through the ingestion of apoptotic cells by macrophages and skewed activation, for example [42], [66].

Hoarau et al. reported high plasma concentrations of IL-12 and IFN-α mRNA in blood mononuclear cells after the convalescence phase, in patients with chronic disease, between 6 months and 1 year after infection. In the patients from Singapore, the concentrations of these two cytokines, measured by alternative techniques, peaked in the acute phase and returned to normal levels at 2–3 months, even in patients who still had clinical symptoms. Consistent with the findings of Hoarau et al., Kelvin et al. and Chaaithanya et al. reported high levels of Th1-type cytokines in the blood of patients with chronic disease (see Table 3). It would thus be extremely interesting to obtain data for larger groups of patients, to determine precisely the cytokine profiles associated with chronic disease. These profiles would constitute a powerful prognostic tool, facilitating preventive treatment and relevant targeted immunomodulatory treatment. Thus, despite certain discrepancies, the available studies analyzed here suggest that chronic disease requires a defect in the regulation of inflammation during the acute and convalescence phases. This lack of regulation results in a deleterious inflammatory process that persists for at least 1 year after the first clinical signs.

**Key Papers**

- Sissoko D, Malvy D, Ezzedine K, Renault P, Moscetti F, et al. (2009) Post-epidemic chikungunya disease on Reunion Island: course of rheumatic manifestations and associated factors over a 15-month period. PLoS Negl Trop Dis 3: e389. doi:10.1371/journal.pntd.0000389
- Gardner J, Anraku I, Le TT, Larcher T, Major L, et al. (2010) Chikungunya virus arthritis in adult wild-type mice. J Virol 84: 8021–8032.
- Labadie K, Larcher T, Joubert C, Mannioui A, Delache B, et al. (2010) Chikungunya disease in nonhuman primates involves long-term viral persistence in macrophages. J Clin Invest 120: 894–906.
- Chow A, Her Z, Ong EK, Chen JM, Dimatatac F, et al. (2011) Persistent arthralgia induced by chikungunya virus infection is associated with interleukin-6 and granulocyte macrophage colony-stimulating factor. J Infect Dis 203: 149–157.
- Hoarau JJ, Jaffar Bandjee MC, Trotot PK, Das T, Li-Pat-Yuen G, et al. (2010) Persistent chronic inflammation and infection by chikungunya arthritogenic alphavirus in spite of a robust host immune response. J Immunol 184: 5914–5927.

**Key Learning Points**

- Chronic signs of chikungunya disease.
- What animal models tell about chikungunya virus persistence.
- Relationship between inflammatory response to infection and chronic rheumatic syndrome.

## References

[1] Enserink M (2006). Infectious diseases. Massive outbreak draws fresh attention to little-known virus.. Science.

[2] Tsetsarkin K, Higgs S, McGee CE, De Lamballerie X, Charrel RN (2006). Infectious clones of Chikungunya virus (La Reunion isolate) for vector competence studies.. Vector Borne Zoonotic Dis.

[3] Economopoulou A, Dominguez M, Helynck B, Sissoko D, Wichmann O (2009). Atypical Chikungunya virus infections: clinical manifestations, mortality and risk factors for severe disease during the 2005–2006 outbreak on Reunion.. Epidemiol Infect.

[4] Dominguez M, Economopoulou A (2007). Surveillance active des formes émergentes hospitalières de chikungunya. La Réunion, avril 2005-mars 2006.

[5] Borgherini G, Poubeau P, Jossaume A, Gouix A, Cotte L (2008). Persistent arthralgia associated with Chikungunya virus: a study of 88 adult patients on Reunion Island.. Clin Infect Dis.

[6] Brighton SW, Simson IW (1984). A destructive arthropathy following Chikungunya virus arthritis–a possible association.. Clin Rheumatol.

[7] Simon F, Parola P, Grandadam M, Fourcade S, Oliver M (2007). Chikungunya infection: an emerging rheumatism among travelers returned from Indian Ocean islands. Report of 47 cases.. Medicine (Baltimore).

[8] Sissoko D, Malvy D, Ezzedine K, Renault P, Moscetti F (2009). Post-epidemic chikungunya disease on Reunion Island: course of rheumatic manifestations and associated factors over a 15-month period.. PLoS Negl Trop Dis.

[9] Fourie ED, Morrison JG (1979). Rheumatoid arthritic syndrome after chikungunya fever.. S Afr Med J.

[10] Delogu I, de Lamballerie X (2011). Chikungunya disease and chloroquine treatment.. J Med Virol.

[11] de Lamballerie X, Ninove L, Charrel RN (2009). Antiviral treatment of chikungunya virus infection.. Infect Disord Drug Targets.

[12] Gould EA, Coutard B, Malet H, Morin B, Jamal S (2010). Understanding the alphaviruses: Recent research on important emerging pathogens and progress towards their control.. Antiviral Res.

[13] Herrero L, Nelson M, Bettadapura J, Gahan ME, Mahalingam S (2011). Applications of animal models of infectious arthritis in drug discovery: a focus on alphaviral disease.. Curr Drug Targets.

[14] Pialoux G, Gauzere BA, Jaureguiberry S, Strobel M (2007). Chikungunya, an epidemic arbovirosis.. Lancet Infect Dis.

[15] Murhekar MV, Manickam P, Kumar RM, Ganesakumar SR, Ramachandran V (2011). Treatment practices & laboratory investigations during chikungunya outbreaks in South India.. Indian J Med Res.

[16] Manimunda SP, Sugunan AP, Rai SK, Vijayachari P, Shriram AN (2010). Outbreak of chikungunya fever, Dakshina Kannada District, South India, 2008.. Am J Trop Med Hyg.

[17] Gerardin P, Guernier V, Perrau J, Fianu A, Le Roux K (2008). Estimating Chikungunya prevalence in La Reunion Island outbreak by serosurveys: two methods for two critical times of the epidemic.. BMC Infect Dis.

[18] Moro ML, Gagliotti C, Silvi G, Angelini R, Sambri V (2010). Chikungunya virus in North-Eastern Italy: a seroprevalence survey.. Am J Trop Med Hyg.

[19] Chopra A, Anuradha V, Ghorpade R, Saluja M (2011). Acute Chikungunya and persistent musculoskeletal pain following the 2006 Indian epidemic: a 2-year prospective rural community study.. Epidemiol Infect.

[20] Sissoko D, Moendandze A, Malvy D, Giry C, Ezzedine K (2008). Seroprevalence and risk factors of chikungunya virus infection in Mayotte, Indian Ocean, 2005–2006: a population-based survey.. PLoS ONE.

[21] Sergon K, Njuguna C, Kalani R, Ofula V, Onyango C (2008). Seroprevalence of Chikungunya virus (CHIKV) infection on Lamu Island, Kenya, October 2004.. Am J Trop Med Hyg.

[22] Sergon K, Yahaya AA, Brown J, Bedja SA, Mlindasse M (2007). Seroprevalence of Chikungunya virus infection on Grande Comore Island, union of the Comoros, 2005.. Am J Trop Med Hyg.

[23] Gerardin P, Barau G, Michault A, Bintner M, Randrianaivo H (2008). Multidisciplinary prospective study of mother-to-child chikungunya virus infections on the island of La Reunion.. PLoS Med.

[24] Panning M, Grywna K, van Esbroeck M, Emmerich P, Drosten C (2008). Chikungunya fever in travelers returning to Europe from the Indian Ocean region, 2006.. Emerg Infect Dis.

[25] Gibney KB, Fischer M, Prince HE, Kramer LD, St George K (2011). Chikungunya fever in the United States: a fifteen year review of cases.. Clin Infect Dis.

[26] Mizuno Y, Kato Y, Takeshita N, Ujiie M, Kobayashi T (2010). Clinical and radiological features of imported chikungunya fever in Japan: a study of six cases at the National Center for Global Health and Medicine.. J Infect Chemother.

[27] Manimunda SP, Vijayachari P, Uppoor R, Sugunan AP, Singh SS (2010). Clinical progression of chikungunya fever during acute and chronic arthritic stages and the changes in joint morphology as revealed by imaging.. Trans R Soc Trop Med Hyg.

[28] Chopra A, Anuradha V, Lagoo-Joshi V, Kunjir V, Salvi S (2008). Chikungunya virus aches and pains: an emerging challenge.. Arthritis Rheum.

[29] Parola P, de Lamballerie X, Jourdan J, Rovery C, Vaillant V (2006). Novel chikungunya virus variant in travelers returning from Indian Ocean islands.. Emerg Infect Dis.

[30] Couderc T, Chretien F, Schilte C, Disson O, Brigitte M (2008). A mouse model for Chikungunya: young age and inefficient Type-I interferon signaling are risk factors for severe disease.. PLoS Pathog.

[31] Gardner J, Anraku I, Le TT, Larcher T, Major L (2010). Chikungunya virus arthritis in adult wild-type mice.. J Virol.

[32] Morrison TE, Oko L, Montgomery SA, Whitmore AC, Lotstein AR (2011). A mouse model of chikungunya virus-induced musculoskeletal inflammatory disease: evidence of arthritis, tenosynovitis, myositis, and persistence.. Am J Pathol.

[33] Rulli NE, Rolph MS, Srikiatkhachorn A, Anantapreecha S, Guglielmotti A (2011). Protection from arthritis and myositis in a mouse model of acute chikungunya virus disease by bindarit, an inhibitor of monocyte chemotactic protein-1 (MCP-1) Synthesis.. J Infect Dis.

[34] Ziegler SA, Lu L, da Rosa AP, Xiao SY, Tesh RB (2008). An animal model for studying the pathogenesis of chikungunya virus infection.. Am J Trop Med Hyg.

[35] Labadie K, Larcher T, Joubert C, Mannioui A, Delache B (2010). Chikungunya disease in nonhuman primates involves long-term viral persistence in macrophages.. J Clin Invest.

[36] Roques P, Gras G, Labadie K, Larcher T, Cherel Y (2011). Chikungunya virus infection involved monocytes and during chronic phase of the disease persisted in tissue macrophages..

[37] Her Z, Malleret B, Chan M, Ong EK, Wong SC (2010). Active infection of human blood monocytes by Chikungunya virus triggers an innate immune response.. J Immunol.

[38] Rinaldo CR, Overall JC, Glasgow LA (1975). Viral replication and interferon production in fetal and adult ovine leukocytes and spleen cells.. Infect Immun.

[39] Sourisseau M, Schilte C, Casartelli N, Trouillet C, Guivel-Benhassine F (2007). Characterization of reemerging chikungunya virus.. PLoS Pathog.

[40] Solignat M, Gay B, Higgs S, Briant L, Devaux C (2009). Replication cycle of chikungunya: a re-emerging arbovirus.. Virology.

[41] Hoarau JJ, Jaffar Bandjee MC, Trotot PK, Das T, Li-Pat-Yuen G (2010). Persistent chronic inflammation and infection by Chikungunya arthritogenic alphavirus in spite of a robust host immune response.. J Immunol.

[42] Krejbich-Trotot P, Denizot M, Hoarau JJ, Jaffar-Bandjee MC, Das T (2011). Chikungunya virus mobilizes the apoptotic machinery to invade host cell defenses.. FASEB J.

[43] Krejbich-Trotot P, Gay B, Li-Pat-Yuen G, Hoarau JJ, Jaffar-Bandjee MC (2011). Chikungunya triggers an autophagic process which promotes viral replication.. Virol J.

[44] Chow A, Her Z, Ong EK, Chen JM, Dimatatac F (2011). Persistent arthralgia induced by Chikungunya virus infection is associated with interleukin-6 and granulocyte macrophage colony-stimulating factor.. J Infect Dis.

[45] Wauquier N, Becquart P, Nkoghe D, Padilla C, Ndjoyi-Mbiguino A (2011). The acute phase of chikungunya virus infection in humans is associated with strong innate immunity and T CD8 cell activation.. J Infect Dis.

[46] Kelvin AA, Banner D, Silvi G, Moro ML, Spataro N (2011). Inflammatory cytokine expression is associated with chikungunya virus resolution and symptom severity.. PLoS Negl Trop Dis.

[47] Borgherini G, Poubeau P, Staikowsky F, Lory M, Le Moullec N (2007). Outbreak of chikungunya on Reunion Island: early clinical and laboratory features in 157 adult patients.. Clin Infect Dis.

[48] Chaaitanya IK, Muruganandam N, Sundaram SG, Kawalekar O, Sugunan AP (2011). Role of proinflammatory cytokines and chemokines in chronic arthropathy in CHIKV infection.. Viral Immunol.

[49] Chirathaworn C, Rianthavorn P, Wuttirattanakowit N, Poovorawan Y (2010). Serum IL-18 and IL-18BP levels in patients with Chikungunya virus infection.. Viral Immunol.

[50] Boyd TD, Bennett SP, Mori T, Governatori N, Runfeldt M (2010). GM-CSF upregulated in rheumatoid arthritis reverses cognitive impairment and amyloidosis in Alzheimer mice.. J Alzheimers Dis.

[51] Cornish AL, Campbell IK, McKenzie BS, Chatfield S, Wicks IP (2009). G-CSF and GM-CSF as therapeutic targets in rheumatoid arthritis.. Nat Rev Rheumatol.

[52] Porcheray F, Viaud S, Rimaniol AC, Leone C, Samah B (2005). Macrophage activation switching: an asset for the resolution of inflammation.. Clin Exp Immunol.

[53] Ogilvie P, Bardi G, Clark-Lewis I, Baggiolini M, Uguccioni M (2001). Eotaxin is a natural antagonist for CCR2 and an agonist for CCR5.. Blood.

[54] Shintani Y, Aoki H, Nishihara M, Ohno S, Furusho A (2011). Hepatocyte growth factor promotes an anti-inflammatory cytokine profile in human abdominal aortic aneurysm tissue.. Atherosclerosis.

[55] Lidbury BA, Rulli NE, Suhrbier A, Smith PN, McColl SR (2008). Macrophage-derived proinflammatory factors contribute to the development of arthritis and myositis after infection with an arthrogenic Alphavirus.. J Infect Dis.

[56] Rulli NE, Guglielmotti A, Mangano G, Rolph MS, Apicella C (2009). Amelioration of alphavirus-induced arthritis and myositis in a mouse model by treatment with bindarit, an inhibitor of monocyte chemotactic proteins.. Arthritis Rheum.

[57] Sugiura T, Kawaguchi Y, Soejima M, Katsumata Y, Gono T (2010). Increased HGF and c-Met in muscle tissues of polymyositis and dermatomyositis patients: beneficial roles of HGF in muscle regeneration.. Clin Immunol.

[58] Kamimoto M, Mizuno S, Nakamura T (2009). Reciprocal regulation of IL-6 and IL-10 balance by HGF via recruitment of heme oxygenase-1 in macrophages for attenuation of liver injury in a mouse model of endotoxemia.. Int J Mol Med.

[59] Coudriet GM, He J, Trucco M, Mars WM, Piganelli JD (2010). Hepatocyte growth factor modulates interleukin-6 production in bone marrow derived macrophages: implications for inflammatory mediated diseases.. PLoS ONE.

[60] Bendinelli P, Matteucci E, Dogliotti G, Corsi MM, Banfi G (2010). Molecular basis of anti-inflammatory action of platelet-rich plasma on human chondrocytes: mechanisms of NF-kappaB inhibition via HGF.. J Cell Physiol.

[61] Schaible HG, von Banchet GS, Boettger MK, Brauer R, Gajda M (2010). The role of proinflammatory cytokines in the generation and maintenance of joint pain.. Ann N Y Acad Sci.

[62] Alonzi T, Fattori E, Lazzaro D, Costa P, Probert L (1998). Interleukin 6 is required for the development of collagen-induced arthritis.. J Exp Med.

[63] Klareskog L, Catrina AI, Paget S (2009). Rheumatoid arthritis.. Lancet.

[64] Biswas P, Poli G, Kinter AL, Justement JS, Stanley SK (1992). Interferon gamma induces the expression of human immunodeficiency virus in persistently infected promonocytic cells (U1) and redirects the production of virions to intracytoplasmic vacuoles in phorbol myristate acetate-differentiated U1 cells.. J Exp Med.

[65] Dhawan S, Heredia A, Wahl LM, Epstein JS, Meltzer MS (1995). Interferon-gamma-induced downregulation of CD4 inhibits the entry of human immunodeficiency virus type-1 in primary monocytes.. Pathobiology.

[66] Fadok VA, Bratton DL, Konowal A, Freed PW, Westcott JY (1998). Macrophages that have ingested apoptotic cells in vitro inhibit proinflammatory cytokine production through autocrine/paracrine mechanisms involving TGF-beta, PGE2, and PAF.. J Clin Invest.

[67] Pialoux G, Gauzere BA, Strobel M (2006). [Chikungunya virus infection: review through an epidemic].. Med Mal Infect.

[68] Tournebize P, Charlin C, Lagrange M (2009). [Neurological manifestations in Chikungunya: About 23 cases collected in Reunion Island.].. Rev Neurol (Paris).

[69] Powers AM (2009). A global overview of chikungynya virus problem. Challenges and insights towards understanding the reemergence of Chikungunya.

[70] Rampal, Sharda M, Meena H (2007). Neurological complications in Chikungunya fever.. J Assoc Physicians India.

[71] Lakshmi V, Neeraja M, Subbalaxmi MV, Parida MM, Dash PK (2008). Clinical features and molecular diagnosis of Chikungunya fever from South India.. Clin Infect Dis.

[72] Inamadar AC, Palit A, Sampagavi VV, Raghunath S, Deshmukh NS (2008). Cutaneous manifestations of chikungunya fever: observations made during a recent outbreak in south India.. Int J Dermatol.

[73] Mishra K, Rajawat V (2008). Chikungunya-induced genital ulcers.. Indian J Dermatol Venereol Leprol.

[74] Mittal A, Mittal S, Bharati MJ, Ramakrishnan R, Saravanan S (2007). Optic neuritis associated with chikungunya virus infection in South India.. Arch Ophthalmol.

[75] SajithKumar R (2009). Chikungunya in India. Challenges and insights towards understanding the reemergence of Chikungunya.

[76] Manimunda SP, Mavalankar D, Bandyopadhyay T, Sugunan AP (2011). Chikungunya epidemic-related mortality.. Epidemiol Infect.

[77] Win MK, Chow A, Dimatatac F, Go CJ, Leo YS (2010). Chikungunya fever in Singapore: Acute clinical and laboratory features, and factors associated with persistent arthralgia.. J Clin Virol.

[78] Ayu SM, Lai LR, Chan YF, Hatim A, Hairi NN (2010). Seroprevalence survey of Chikungunya virus in Bagan Panchor, Malaysia..

[79] Chua KB (2010). Epidemiology of chikungunya in Malaysia: 2006–2009.. Med J Malaysia.

[80] Chua HH, Abdul Rashid K, Law WC, Hamizah A, Chem YK (2010). A fatal case of chikungunya virus infection with liver involvement.. Med J Malaysia.

[81] Singh SS, Manimunda SP, Sugunan AP, Sahina, Vijayachari P (2008). Four cases of acute flaccid paralysis associated with chikungunya virus infection.. Epidemiol Infect.

[82] Le Bomin A, Hebert JC, Marty P, Delaunay P (2008). [Confirmed chikungunya in children in Mayotte. Description of 50 patients hospitalized from February to June 2006].. Med Trop (Mars).

[83] Moiton MP, Jaffar Bandjee MC, Gay F (2008). Acute and chronic rheumatic symptoms of chikungunya in adults: acquired knowledge during the Reunion Island outbreak, France (2005–2006).. Bulletin d'épidémiologie hebdomadaire.

